# Enhanced recovery after surgery (ERAS) for vascular surgery: an evidence map and scoping review

**DOI:** 10.1186/s13643-023-02324-z

**Published:** 2023-09-14

**Authors:** Eric A. Apaydin, Karen Woo, Julia Rollison, Sangita Baxi, Aneesa Motala, Susanne Hempel

**Affiliations:** 1 Southern California Evidence Review Center, Keck School of Medicine, University of Southern California, Los Angeles, CA USA; 2 RAND Health Care, RAND Corporation, Santa Monica, CA USA; 3 Center for the Study of Healthcare Innovation, Implementation, and Policy, VA Greater Los Angeles Healthcare System, Los Angeles, CA USA; 4 Department of Surgery, David Geffen School of Medicine, University of California, Los Angeles, Los Angeles, CA USA; 5 RAND Health Care, RAND Corporation, Arlington, VA USA

**Keywords:** Enhanced recovery, Surgery, Vascular, Evidence map

## Abstract

**Background:**

Enhanced recovery after surgery (ERAS) interventions aim to improve patient outcomes. Vascular surgery patients have unique requirements and it is unclear which ERAS interventions are supported by an evidence base.

**Methods:**

We conducted a scoping review to identify ERAS randomized controlled trials (RCTs) published in the biomedical or nursing literature. We assessed interventions for applicability to vascular surgery and differentiated interventions given at preadmission, preoperative, intraoperative, and postoperative surgery stages. We documented the research in an evidence map.

**Results:**

We identified 76 relevant RCTs. Interventions were mostly administered in preoperative (23 RCTs; 30%) or intraoperative surgery stages (35 RCTs; 46%). The majority of studies reported mortality outcomes (44 RCTs; 58%), but hospital (27 RCTs; 35%) and intensive care unit (9 RCTs; 12%) length of stay outcomes were less consistently described.

**Conclusion:**

The ERAS evidence base is growing but contains gaps. Research on preadmission interventions and more consistent reporting of key outcomes is needed.

**Supplementary Information:**

The online version contains supplementary material available at 10.1186/s13643-023-02324-z.

## Introduction

Enhanced recovery after surgery (ERAS) consists of interventions designed to support patients recovering from surgery throughout the continuum of care [1]. Improved recovery after surgery benefits patients, practitioners, and healthcare delivery organizations [2]. However, little is known about the applicability ERAS interventions to vascular surgery [3]. Vascular surgery, which includes a wide range of treatments that encompass major open operations and less invasive endovascular procedures, may benefit from ERAS approaches.

To better understand this literature, we conducted a scoping review and created an evidence map of randomized controlled trials (RCTs) for ERAS interventions, mapped by surgical stage, that evaluate key patient-centered outcomes (mortality and length of stay). The evidence map [4] approach allows readers to view the existing evidence base in one figure and identify key research gaps.

## Methods

As part of a larger project on ERAS, we searched PubMed and the Cumulative Index to Nursing and Allied Health Literature for published literature, and searched ClinicalTrials.gov for clinical trial records from inception to March 2023 (search strategies shown in Supplementary Material 1). Citations and full text publications were screened by experienced literature reviewers using predetermined eligibility criteria (full criteria shown in Supplementary Material 2). Notably, we restricted to RCTs, a study design that allows strong evidence statements. Abstracted items are also listed in Supplementary Material 2. Results were plotted as an evidence map using data visualization software (R Studio; R Studio Public Benefit Corporation; Boston, MA). All data analyzed in this manuscript is available in Table 1. This study did not involve human subjects and was therefore exempt from RAND Human Subjects Protection Committee review. Our full report on ERAS interventions for vascular surgery, with a search from inception to July 2019, is available on the Patient-Centered Outcomes Research Institute (PCORI) website [5].

## Results

The search identified 4,483 citations, and we included 79 RCTs of ERAS interventions for vascular surgery (evidence table: Table 1) [6–84].

**Table 1:** Evidence table

Citation	Stage	Mortality	LOS	ICU LOS
Ali 2007 [6]	Intraoperative	Measured	Measured	Measured
Baldwin 1994 [7]	Postoperative	Measured		
Barlow 1989 [8]	Preoperative			
Belch 1980 [9]	Preoperative	Measured		
Bender 1997 [10]	Preoperative	Measured		
Berlauk 1991 [11]	Preoperative	Measured		Measured
Bille-Brahe 1980 [12]	Postoperative			
Bode 1996 [13]	Intraoperative	Measured	Measured	Measured
Bohner 2002 [14]	Postoperative	Measured	Measured	Measured
Bolliger 2007 [15]	Preoperative	Measured		
Bonazzi 2002 [16]	Intraoperative	Measured	Measured	
Brady 2005 [17]	Intraoperative	Measured	Measured	
Christopherson 1996 [18]	Intraoperative	Measured		
Cook 1986 [19]	Intraoperative	Measured		
Dorman 1995 [20]	Intraoperative			
Durazzo 2004 [21]	Preoperative	Measured		
Earnshaw 1989 [22]	Postoperative			
Farkas 1993 [23]	Multi-stage	Measured		
Fleisher 2005 [24]	Preoperative	Measured		
Fleron 2003 [25]	Intraoperative	Measured		
Forster 2006 [26]	Postoperative			
Fourneau 2006 [27]	Intraoperative		Measured	
Frank 1992 [28]	Intraoperative			
Friedman 1996 [29]	Postoperative		Measured	Measured
Gonzalez-Fajardo 2009 [30]	Postoperative		Measured	
Gouaillier-Vulcain 2015 [31]	Intraoperative			
Hall 1998 [32]	Preoperative			
Hasselgren 1984 [33]	Preoperative			
Healy 2015 [34]	Intraoperative	Measured	Measured	Measured
Kaiser 1978 [35]	Intraoperative			
Kavakli 2019 [36]	Intraoperative		Measured	Measured
Kouvelos 2013 [37]	Preoperative			
Krog 2017 [38]	Intraoperative	Measured	Measured	
Kucukakin 2010 [39]	Intraoperative			
Kwon 2018 [40]	Postoperative		Measured	
Lee 2017 [41]	Postoperative	Measured	Measured	
Lindholm 2013 [42]	Intraoperative	Measured	Measured	
Linni 2012 [43]	Preoperative	Measured	Measured	
Lundorff 1999 [44]	Intraoperative			
Lunen 2018 [45]	Preoperative	Measured	Measured	Measured
Marroni 1999 [46]	Preoperative	Measured		
Martin 1982 [47]	Intraoperative			
Miller 1994 [48]	Postoperative	Measured	Measured	
Monsel 2016 [49]	Postoperative			
Mouren 1989 [50]	Intraoperative			
Muehling 2009 [51]	Multi-stage	Measured	Measured	Measured
Murphy 2014 [52]	Intraoperative	Measured	Measured	
Nesek-Adam 2012 [53]	Intraoperative			
Nevelsteen 1991 [54]	Preoperative			
Niemi 2006 [55]	Intraoperative			
Norgen 2004 [56]	Intraoperative	Measured		
Norris 2001 [57]	Multi-stage	Measured	Measured	
Oliver 2006 [58]	Intraoperative	Measured		
Ozaki 2015 [59]	Postoperative			
Partridge 2017 [60]	Preoperative	Measured	Measured	
Pitt 1980 [61]	Intraoperative	Measured		
Pleger 2018 [62]	Postoperative		Measured	
Poldermans 1999 [63]	Intraoperative	Measured		
Reinhart 1989 [64]	Intraoperative	Measured		
Renghi 2013 [65]	Intraoperative		Measured	
Risberg 1995 [66]	Preoperative	Measured		
Roizen 1980 [67]	Preoperative			
Salzmann 1983 [68]	Intraoperative			
Schouten 2009 [69]	Intraoperative	Measured		
Soliman 2016 [70]	Preoperative	Measured	Measured	
Sprung 2000 [71]	Intraoperative			
Stuhmeier 1996 [72]	Preoperative	Measured		
Subramaniam 2009 [73]	Intraoperative	Measured	Measured	
Swinnen 2010 [74]	Intraoperative			
Thomas 2016 [75]	Preoperative	Measured	Measured	
Turner 2008 [76]	Preoperative	Measured	Measured	Measured
Turtianien 2012 [77]	Postoperative	Measured	Measured	
Van der Linden 2010 [78]	Intraoperative	Measured		
Vierhout 2014 [79]	Postoperative			
Vukovic 2012 [80]	Postoperative			
Weed 2017 [81]	Preoperative		Measured	
Worning 1986 [82]	Preoperative			
Yang 2006 [83]	Intraoperative	Measured	Measured	
Ziegler 1997 [84]	Preoperative	Measured		Measured

Abbreviations: *ICU *Intensive care unit, *LOS *Length of stay

**Fig. 1:**
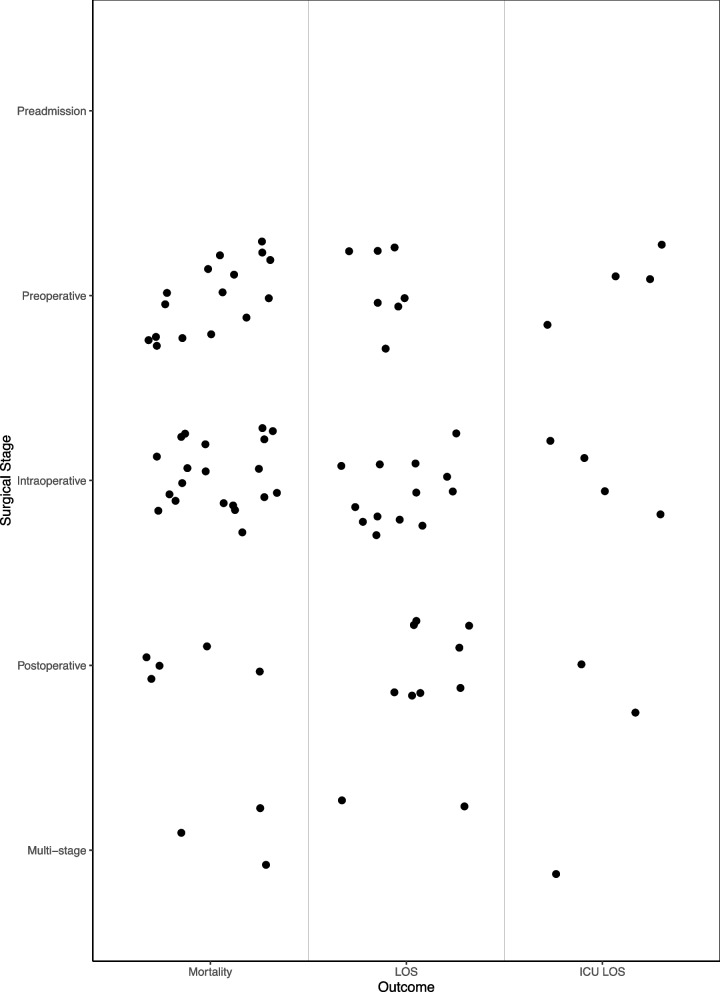
Evidence map of reported outcomes in included ERAS studies by surgical stage Abbreviations: *ICU* Intensive care unit, *LOS* Length of stay

Figure 1 documents the evidence base and distribution of evidence across treatment stages and treatment outcomes. Of the evaluated interventions, 24 (30%) were preoperative, 36 (46%) were intraoperative, 16 (20%) were postoperative, and 3 (4%) were multi-stage. We did not identify any preadmission interventions. Forty-four RCTs (56%) reported mortality outcomes (Fig. 1), and of these interventions, 16 (36%) were preoperative, 20 (45%) were intraoperative, 5 (11%) were postoperative, and 3 (7%) were multi-stage. Length of stay outcomes were reported in 30 RCTs (38%), which evaluated ERAS interventions administered preoperatively (7 RCTs; 23%), intraoperatively (13 RCTS; 43%), postoperatively (8 RCTs; 27%), and across multiple surgical stages (2 RCTs; 7%). Length of stay in intensive care units (ICUs) was measured least frequently, in only 11 RCTs (14%). Of interventions in these studies, 4 (36%) were preoperative, 4 (36%) were intraoperative, 2 (18%) were postoperative, and 1 (9%) was multi-stage.

## Discussion

We identified a substantial body of evidence of ERAS interventions for vascular surgery. Our analysis utilized an evidence map to categorize the available research on RCTs reporting mortality and length of stay outcomes for these interventions by surgical stage, a strategy not previously employed by other reviews. The map shows that existing research has primarily addressed pre- or intraoperative, rather than preadmission, surgical stages. In addition, while more than half of studies reported on mortality, information on hospital or ICU length of stay remains sparse.

A recent review [85] of 19 RCTs and observational studies of ERAS interventions for vascular surgery found that ERAS interventions reduced length of stay by 3.5 days across five studies. The review did not report pooled effects for mortality outcomes. A comprehensive systematic review and meta-analysis of the effects of ERAS interventions in the extant literature is needed to better estimate treatment effects.

Additional primary research on ERAS interventions is also needed. The existing evidence base lacks studies of preadmission ERAS interventions, and mortality and length of stay outcomes were not consistently reported. Future primary research should aim to study preadmission interventions and consistently measure and report mortality and length of stay outcomes.

ERAS research is rapidly growing and an evidence base for vascular surgery is also emerging. Our evidence map clearly outlines research gaps, including a lack of research on ERAS interventions at all surgery stages and the sparseness of information on key outcomes. ERAS interventions hold promise to improve patient recovery after vascular surgery and further applications should be explored.

### Supplementary Information

**Additional file 1: Supplementary Material 1.** Search strategies. **Supplementary Material 2.** Eligibility criteria and data abstraction methods.

## Data Availability

All data analyzed in this manuscript is available in Table 1.
