# Supplementary material for: Enhanced recovery after surgery (ERAS) for vascular surgery: an evidence map and scoping review
Source: Syst Rev. 2023 Sep 14;12:162. doi: 10.1186/s13643-023-02324-z (PMC10500918; doi:10.1186/s13643-023-02324-z)
Supplement: Supplementary file 1 — Additional file 1: Supplementary Material 1. Search strategies. Supplementary Material 2. Eligibility criteria and data abstraction methods. [file 13643_2023_2324_MOESM1_ESM.docx]

**Supplementary Material**

**Supplementary Material 1. Search strategies**

*PubMed*

((“enhanced recovery after surgery”[title/abstract] OR “ERAS” [title/abstract]

OR “enhanced recovery pathway”[title/abstract] OR post-operative recovery[title/abstract] OR “enhanced recovery pathways”[title/abstract] OR enhanced recovery protocol[title/abstract] OR (“enhanced recovery” AND “multimodal pathway”[title/abstract]) OR (“enhanced recovery” AND “multimodal pathways”[title/abstract]) OR (“enhanced recovery” AND surger*[title/abstract]) OR (“ERAS” AND “multimodal pathway”[title/abstract]) OR (“ERAS” AND “multimodal pathways”[title/abstract]) OR (recovery AND perioperative pathways[title/abstract]))

OR (“enhanced recovery” OR fast track recovery[title/abstract] OR “accelerated recovery” OR “early recovery” OR “early discharge” OR accelerated recovery[title/abstract] OR rapid recovery[title/abstract] OR “early mobilization” OR vascular surgery[title/abstract]) AND (“care pathway”[title/abstract] OR “care pathways”[title/abstract]

OR (pre-admission AND intervention OR procedure) OR ((“patient education” OR “patient screening” OR patient expectation setting[title/abstract] OR “pre-conditioning” OR pre- habilitation” OR pre-operative exercise) AND (tobacco screening[title/abstract] OR alcohol screening[title/abstract] OR nutritional deficiency screening[title/abstract] OR frailty screening[title/abstract] OR anemia screening[title/abstract], OR diabetes

screening[title/abstract] OR coronary artery disease screening[title/abstract] OR nutritional optimization[title/abstract]) OR (“medical screening” OR “medical optimization”[title/abstract]) OR “preadmission screening”[title/abstract] OR preadmission nutrition*[title/abstract] “cognitive impairment screening”[title/abstract] OR “antiplatelet planning”[ALL FIELDS] OR (anticoagulation AND planning[title/abstract])

OR (“Preoperative intervention” OR “pre-operative intervention”[title/abstract]) OR (“Preoperative interventions” OR “pre-operative interventions”[title/abstract] OR preoperative protocol*[title/abstract] OR pre-operative protocol*[title/abstract] OR “preoperative preparation”[title/abstract] OR “pre-operative preparation”)

OR ((“limited fasting”[title/abstract] OR “recommended fasting” [title/abstract] OR (“carbohydrate loading”[title/abstract] AND diabetes[title/abstract]) OR “pre-emptive analgesia”[title/abstract] OR “Antiemetic Prophylaxis”[title/abstract] OR anti-emetic prophylaxis[title/abstract] OR antimicrobial prophylaxis[title/abstract] OR antimicrobial shower OR antimicrobial soap) AND (pre-operative OR preoperative))

OR “intraoperative intervention”[title/abstract] OR “intraoperative interventions”[title/abstract] OR intraoperative management[title/abstract] OR perioperative intervention OR perioperative interventions OR perioperative protocol*[title/abstract] OR “anesthetic plan”[title/abstract] OR “anesthesia plan”[title/abstract] OR “regional anesthesia”[title/abstract] OR “neuraxial anesthesia”

OR (“post operative interventions” OR “post operative interventions”[title/abstract] OR “post operative” protocol*[title/abstract] OR post-operative protocol*[title/abstract] OR postoperative protocol[title/abstract]) OR “patient warming”[title/abstract] OR (“fluid management strategy” OR “fluid management strategies”[title/abstract]) OR (“multimodal analgesia” OR “opioid sparing analgesia” OR “multi modal analgesia”[title/abstract]) OR (“opioid minimization”[Title/Abstract]) AND (strategy[Title/Abstract] OR strategies[Title/Abstract]) OR

“drain management”[title/abstract] OR surgical drain[title/abstract] OR “line management”[title/abstract]
OR early “drain removal”[title/abstract] OR early “line removal”[title/abstract] OR “foley catheter”[title/abstract] OR nasogastric tube*[title/abstract] OR pulmonary toilet OR “early mobilization strategies”[title/abstract] OR “early mobilization”[title/abstract] OR (“diet regimen” OR “diet regimens”)[title/abstract] OR (“bowel regimen” OR “bowel regimens” OR anti- emetic)[title/abstract] OR ((plan OR plans OR planning) AND patient discharge[MESH]) OR (“discharge plan” OR “discharge plans” OR “discharge planning”)[title/abstract]))
AND
(“surgical procedure”[title/abstract] OR “surgical procedures”[title/abstract] OR Surgical Procedures, Operative[Majr] OR “vascular surgery”[title/abstract] OR “endovascular surgery” [title/abstract])
AND
(systematic review [publication type] OR (“clinical trial” OR “clinical trials”[title/abstract]) OR “Cohort Studies”[Majr] OR (“cohort study” OR “cohort studies”[title/abstract]) OR “case series”[title/abstract] OR “pre post”[title/abstract] OR “before after”[title/abstract] OR Controlled Before-After Studies[Majr] OR “time series”[title/abstract] OR (“randomized controlled trial” OR “randomized control trials”[title/abstract]) OR Randomized Controlled Trial [Publication Type] OR Randomized Controlled Trials as Topic[Majr] OR (“clinical trial” OR “clinical trials”[title/abstract]) OR Clinical Trial [Publication Type] OR Clinical Trials as Topic[Majr])

*CINAHL*

“enhanced recovery after surgery” OR “ERAS” OR “enhanced recovery pathway” OR post- operative recovery OR “enhanced recovery pathways” OR enhanced recovery protocol OR (“enhanced recovery” AND “multimodal pathway”) OR (“enhanced recovery” AND “multimodal pathways”) OR (“enhanced recovery” AND surger*) OR (“ERAS” AND “multimodal pathway”) OR (“ERAS” AND “multimodal pathways”) OR (recovery AND “perioperative pathways”)

OR (“enhanced recovery” OR fast track recovery OR “accelerated recovery” OR “early recovery” OR “early discharge” OR “rapid recovery” OR “early mobilization” OR “vascular surgery”) AND (“care pathway” OR “care pathways” OR (“pre-admission” AND intervention OR procedure)
OR (“patient education” OR “patient screening” OR “patient expectation setting” OR “pre- conditioning” OR “pre-habilitation” OR “pre-operative exercise”) AND (“tobacco screening” OR “alcohol screening” OR “nutritional deficiency screening” OR “frailty screening” OR “anemia screening” OR “diabetes screening” OR “coronary artery disease screening” OR “nutritional optimization”) OR “medical screening” OR “medical optimization” OR “preadmission screening” OR “preadmission nutrition*” OR “cognitive impairment screening” OR “antiplatelet planning” OR (anticoagulation AND planning)
OR “Preoperative intervention” OR “pre-operative intervention” OR “Preoperative interventions” OR “pre-operative interventions” OR “preoperative protocol*” OR “pre-operative protocol*” OR “preoperative preparation” OR “pre-operative preparation”)
OR (“limited fasting” OR “recommended fasting” OR “carbohydrate loading” AND diabetes) OR (“pre-emptive analgesia” OR “Antiemetic Prophylaxis” OR “anti-emetic prophylaxis” OR“antimicrobial prophylaxis” OR “antimicrobial shower” OR “antimicrobial soap”) AND (“pre- operative” OR preoperative)
OR “intraoperative intervention” OR “intraoperative interventions” OR “intraoperative management” OR “perioperative intervention” OR “perioperative interventions” OR “perioperative protocol*” OR “anesthetic plan” OR “anesthesia plan” OR “regional anesthesia” OR “neuraxial anesthesia”

OR “post operative interventions” OR “post operative interventions” OR “post operative” protocol* OR “post-operative protocol*” OR “postoperative protocol” OR “patient warming” OR “fluid management strategy” OR “fluid management strategies”
OR (“multimodal analgesia” OR “multi modal analgesia” OR “opioid minimization” OR “opioid sparing analgesia”) AND (strategy OR strategies OR “drain management” OR “surgical drain” OR “line management” OR “early drain removal” OR “early line removal” OR “foley catheter” OR “nasogastric tube*” OR “pulmonary toilet” OR “early mobilization strategies” OR “early mobilization” OR “diet regimen” OR “diet regimens” OR “bowel regimen” OR “bowel regimens” OR “anti-emetic” OR plan OR plans OR planning) AND (“patient discharge” OR “discharge plan” OR “discharge plans” OR “discharge planning”)

AND
“surgical procedure” OR “surgical procedures” OR “vascular surgery” OR “endovascular surgery”
AND
“clinical trial” OR “clinical trials” OR “Cohort Studies” OR “cohort study” OR “case series” OR “pre post” OR “before after” OR “Controlled Before-After Studies” OR “time series” OR “randomized controlled trial” OR “randomized control trials” OR “clinical trial” OR “clinical trials”

*Clinicaltrials.gov*

Topics: ERAS, recovery
Context: Surgery, surgical procedures
Study designs All ongoing studies that have stopped recruiting

**Supplementary Material 2. Eligibility criteria and data abstraction methods**

Eligibility criteria

*Population:* Patients of any age undergoing surgical procedures

*Intervention:* Interventions that aim to enhance recovery after surgery; this includes preadmission ERAS interventions and care pathways (e.g., patient education, screening on tobacco abuse, medical screening and optimization, cognitive impairment screening, antiplatelet and anticoagulation planning), preoperative ERAS interventions (e.g., limited fasting, carbohydrate loading, preemptive analgesia, antiemetic prophylaxis), intraoperative ERAS interventions (e.g., specific surgical techniques, anesthetic plans, fluid management strategies), and postoperative ERAS interventions (e.g., patient warming, fluid management strategies, multimodal analgesia, opioid minimization strategies, drain and line management, early mobilization strategies, diet and bowel regimens, and discharge planning). We included comparisons of vascular and endovascular procedures; we accepted open thoracic, open abdominal, and lower extremity bypass as vascular surgery, but we excluded studies on carotid endarterectomy and nonsurgical vascular procedures (e.g., angioplasty). We also excluded all interventions not directly aimed at patients’ recovery, studies assessing the effects of surgical interventions compared with other alternatives (e.g., vascular bypass vs. lifestyle intervention), interventions not associated with recovery from surgery, and studies comparing 2 surgical interventions without reference to recovery.

*Comparator:* We placed no restrictions on the type of comparator, but either a historic (e.g., pre-ERAS intervention) or concurrent (e.g., control group in a clinical trial) comparator had to be reported. We excluded analytic studies aiming to identify predictors of early recovery.

*Outcomes:* Patient health outcomes relevant to recovery, such as mortality, postoperative complications, functional status, postoperative pain control, postoperative time to regular diet, postoperative time to ambulation, hospital length of stay, discharge disposition, or readmissions. We excluded studies exclusively reporting preoperative or perioperative outcomes, acceptability, feasibility, procedure volume, and physiological or invitro indicators.

*Study design:* Only randomized controlled trials (RCTs) were included. Systematic reviews on vascular surgery were retained for reference mining.

*Setting:* No restrictions.

*Timing:* Interventions from preoperative clinic visit/decision for surgery through 90 days postoperative. Follow-up timing was not restricted.

*Other:* We excluded studies without abstracts and those published in abbreviated form (e.g., conference abstract), as these did not provide sufficient information for this topic brief.

Data abstraction methods

We categorized included studies by intervention administration stage: preadmission (intervention started before admission to hospital/clinic); preoperative (started before operation); intraoperative (started during operation; including operative techniques); postoperative (started after operation); and multi-stage (spanning different care entry points). We abstracted reported data for mortality and length of stay.
